# Robotic-assisted laparoscopic adrenalectomy (RARLA): What advantages and disadvantages compared to retroperitoneal laparoscopic adrenalectomy (RLA)?

**DOI:** 10.3389/fendo.2023.1145820

**Published:** 2023-03-02

**Authors:** Xuwen Li, Song Xiao, Yue Yu, Wei Liu, Haibo Xi, Gongxian Wang, Xiaochen Zhou

**Affiliations:** Department of Urology, The First Affiliated Hospital of Nanchang University, Nanchang, China

**Keywords:** robot-assisted, laparoscopic, adrenalectomy, retroperitoneal, surgery

## Abstract

**Objective:**

To explore the advantages and disadvantages of robot-assisted laparoscopic adrenalectomy compared with retroperitoneal laparoscopic adrenalectomy.

**Methods:**

A total of 101 patients with adrenal tumors who received retroperitoneal laparoscopic adrenalectomy (RLA) (n=75) or robot-assisted laparoscopic adrenalectomy (RARLA) (n=26) in our hospital from January 2021 to December 2021 were retrospectively collected. Patients’ demographics, tumor characteristics, and perioperative indicators were compared. Statistical analysis was performed using t-test for continuous variables and Pearson chi-square test or Fisher’s exact test for categorical variables.

**Results:**

We found that blood loss in the RARLA group was significantly less than that in the RLA group (66.9 ± 35.5 ml vs 91.5 ± 66.1 ml, *p* = 0.020). Gastrointestinal function recovery time in RARLA group was significantly less than that in RLA group (19.9 ± 6.9 hours vs 32.0 ± 9.0 hours, *p* < 0.001). However, the operation time, drainage tube placement time, post-operative hospital stay in the RARLA group were significantly longer compared with the RLA group (149.6 ± 53.4 mins vs 118.7 ± 41.2 mins, *p* = 0.003; 4.9 ± 2.0 days vs 3.6 ± 1.1 days, *p* = 0.004; 6.4 ± 1.8 days vs 4.6 ± 1.6 days, *p* < 0.001). The hospitalization expense in the RARLA group is significantly higher than that in the RLA group (59284 ± 8724 RMB¥ vs 39785 ± 10126 RMB¥, *p* < 0.001). We found that there was no significant difference in the incidence of postoperative complications between the two groups. However, the pathological types of the two groups were significantly different. Patients in the RLA group had a higher proportion of adrenocortical adenoma, while patients in the RARLA group had a higher proportion of pheochromocytoma.

**Conclusion:**

Compared with traditional laparoscopic adrenalectomy, robot-assisted laparoscopic adrenalectomy can significantly reduce intraoperative blood loss and accelerate postoperative gastrointestinal recovery. It is committed to studying how to reduce the hospitalization time and hospitalization cost of RARLA, which can make RARLA more widely used.

## 1 Introduction

Adrenal gland is an important endocrine organ, and adrenal tumor is the most common adrenal disease. Since Gagner et al. (1) first reported successful laparoscopic adrenalectomy in 1992, laparoscopic adrenalectomy has become the gold standard for the treatment of adrenal tumors (2). In recent years, with the development of Da Vinci surgical robot, Da Vinci robot has gradually appeared in urological surgery, such as radical prostatectomy, radical cystectomy, partial nephrectomy and so on (3, 4). Many scholars have also studied the application of robots in adrenalectomy. Some scholars thought that robot-assisted laparoscopy has no obvious advantage in the treatment of adrenal tumors compared with traditional laparoscopy, and its application value is controversial. Karen et al. found that the subjective benefits of robotic surgery include a three-dimensional surgical field of view, an ergonomically comfortable position, and the elimination of tremors in the surgeon. Robot-assisted laparoscopic surgery takes significantly longer, but patient outcomes are similar to laparoscopic techniques (5). However, some scholars found that robot-assisted adrenalectomy can safely and effectively shorten operation time. In addition, it also has the advantages of short hospital stay, less blood loss, and low incidence of postoperative complications compared with laparoscopic adrenalectomy (6). These findings seem to support the use of robotic minimally invasive surgery for adrenal tumors. This study compared and analyzed the effects of Da Vinci robot-assisted laparoscopic and retroperitoneal laparoscopic adrenalectomy (RLA) in the Department of Urology, the First Affiliated Hospital of Nanchang University, and explored the advantages and disadvantages of robot-assisted laparoscopic adrenalectomy (RARLA).

## 2 Materials and methods

### 2.1 Data source and ethics statement

This study was conducted with the approval of the Institutional Review Committee and the Ethics Committee of the First Affiliated Hospital of Nanchang University. We used the hospital database to collect the basic, clinical and pathological information of patients undergoing adrenalectomy.

### 2.2 Patient selection

The patients with adrenal tumors who received retroperitoneal laparoscopic adrenalectomy (RLA) or robot-assisted laparoscopic adrenalectomy (RARLA) in our hospital from January 2021 to December 2021 were retrospectively collected. The patients were included in this study according to the following inclusion criteria: [1] Patients diagnosed as adrenal tumor; [2] Unilateral tumor. The exclusion criteria were as follows: [1] During hospitalization, other operations other than adrenalectomy were performed; [2] accompanied by other serious comorbidity.

### 2.3 Technical considerations

After signing informed consent, patients received retroperitoneal laparoscopic adrenalectomy (RLA) or robot-assisted laparoscopic adrenalectomy (RARLA) (RLA and RARLA were both performed *via* the retroperitoneoscopic approach). All operations were performed by an experienced surgical team.

### 2.4 Variables and endpoints

Variables in the study include demographic characteristics [age, sex, body mass index (BMI)], tumor characteristics (tumor size, tumor site, pathologic type), treatment methods (RLA, RARLA), perioperative results (operation time, blood loss, gastrointestinal function recovery time, complication, drainage tube placement time, postoperative hospital stay), and other variable (hospitalization expense). Drainage tube was placed routinely for patients after adrenalectomy. When the postoperative drainage fluid is less than 30 ml per day, the doctors will consider removing the drainage tube according to the patient ‘s condition.

The end points of this study were to compare the average differences in operation time, blood loss, gastrointestinal function recovery time, complication, drainage tube placement time, postoperative hospital stay and hospitalization expense between RLA group and RARLA group.

### 2.5 Statistical analysis

Means and standard deviations were determined for the normally distributed continuous variables. Categorical variables were presented as frequencies and their proportions. The Student’s t-test was performed for the normally distributed continuous variables. All categorical variables were compared with the Chisquare test. SPSS 26.0 (IBM Corp, Armonk, NY) was utilized for all statistical analysis with a two-sided p value < 0.05 denoting statistical significance.

## 3 Results

According to the inclusion and exclusion criteria, 101 patients were included in this study from January 2021 to December 2021. 75 patients chose to perform retroperitoneal laparoscopic surgery, while only 26 patients chose to perform robot-assisted laparoscopic surgery. No significant differences in terms of age (*p* = 0.471), sex (*p* = 0.668), BMI (*p* = 0.909), and tumor site (*p* = 0.707) was observed between two groups (all *p* > 0.05). However, tumor size in the RARLA group tended to be larger compared with RLA group (4.9 ± 2.9 cm vs 2.4 ± 1.1 cm, *p* < 0.001) (**Table 1**).

**Table 1:** Baseline demographical and clinicopathological characteristics of patients.

Variables	RLA (n = 75)	RARLA (n = 26)	*P*
Age, year, mean (SD)	49.7 (12.9)	47.4 (15.3)	0.471
Sex, n (%)			0.668
Male	44.0 (58.7 %)	14.0 (53.8 %)	
Female	31.0 (41.3 %)	12.0 (46.2 %)	
BMI, kg/m^2^, mean (SD)	24.1 (3.8)	24.0 (3.4)	0.909
Tumor size, cm, mean (SD)	2.4 (1.1)	4.9 (2.9)	< 0.001
Tumor site, n (%)			0.701
Left	35.0 (46.7 %)	11.0 (42.3 %)	
Right	40.0 (53.3 %)	15.0 (57.7 %)	

RLA, Retroperitoneal laparoscopic adrenalectomy; RARLA, Robot assisted laparoscopic adrenalectomy; SD, standard deviation; BMI, body mass index.

We found that blood loss in the RARLA group was significantly less than that in the RLA group (66.9 ± 35.5 ml vs 91.5 ± 66.1 ml, *p* = 0.020). Gastrointestinal function recovery time in RARLA group was significantly less than that in RLA group (19.9 ± 6.9 hours vs 32.0 ± 9.0 hours, *p* < 0.001). However, the operation time, drainage tube placement time, post-operative hospital stay in the RARLA group is significantly longer compared with the RLA group (149.6 ± 53.4 mins vs 118.7 ± 41.2 mins, *p* = 0.003; 4.9 ± 2.0 days vs 3.6 ± 1.1 days, *p* = 0.004; 6.4 ± 1.8 days vs 4.6 ± 1.6 days, *p* < 0.001). The hospitalization expense in the RARLA group is significantly higher than that in the RLA group (59284 ± 8724 RMB¥ vs 39785 ± 10126 RMB¥, *p* < 0.001). We found that there was no significant difference in the incidence of postoperative complications between the two groups (4.0% vs 3.8%). By the way, the four patients with complications in the retrospective cohort were classified as grade I according to the Clavien-Dindo classification. However, the pathological types of the two groups were significantly different. Patients in the RLA group had a higher proportion of adrenocortical adenoma (50.0% vs 88.0%), while patients in the RARLA group had a higher proportion of pheochromocytoma (11.5% vs 5.3%) (**Table 2**).

**Table 2:** Comparison of perioperative and pathological results.

Variables	RLA (n = 75)	RARLA (n = 26)	*P*
Operation time, min, mean (SD)	118.7 (41.2)	149.6 (53.4)	0.003
Blood loss, ml, mean (SD)	91.5 (66.1)	66.9 (35.5)	0.020
Gastrointestinal function recovery time, hour, mean (SD)	32.0 (9.0)	19.9 (6.9)	< 0.001
Complication, n (%)			1.000
Yes	3 (4.0 %)	1 (3.8 %)	
None	72 (96.0 %)	25 (96.2 %)	
Drainage tube placement time, day, mean (SD)	3.6 (1.1)	4.9 (2.0)	0.004
Postoperative hospital stay, day, mean (SD)	4.9 (1.6)	6.4 (1.8)	< 0.001
Hospitalization expense, RMB¥, mean (SD)	39785 (10126)	59284 (8724)	< 0.001
Pathologic types, n (%)			< 0.001
Adrenocortical adenoma	66 (88.0 %)	13 (50.0 %)	
Pheochromocytoma	4 (5.3 %)	3 (11.5 %)	
Others pathologic types	5 (6.7 %)	10 (38.5 %)	

RLA, Retroperitoneal laparoscopic adrenalectomy; RARLA, Robot assisted laparoscopic adrenalectomy; SD, standard deviation; BMI, body mass index.

## 4 Discussion

With the development of minimally invasive technology, laparoscopy is more and more widely used in surgery. Laparoscopic adrenalectomy has become the preferred treatment for most adrenal tumors. Compared with open adrenalectomy, patients have better tolerance to laparoscopic adrenalectomy (7). In 2001, Horgan et al. (8) first used the Da Vinci robotic surgical system to complete unilateral adrenalectomy. They found that robotic surgery was a safe and effective alternative to traditional laparoscopic surgery. With the development of robotic surgery, many medical centers began to perform robot-assisted laparoscopic adrenalectomy, and the safety and effectiveness of this surgical method have been verified (9, 10).

There have been some comparative studies on robot-assisted laparoscopic adrenalectomy and traditional laparoscopic adrenalectomy. Agcaoglu et al. (11) have shown that for larger adrenal tumors, robot-assisted laparoscopic adrenalectomy can shorten the operation time and reduce the probability of conversion to open surgery. Robot-assisted laparoscopic adrenalectomy can be used as the preferred surgical method for larger adrenal tumors. However, in their study, only adrenal tumors larger than 5cm in diameter were included. Karabulut et al. (12) have shown that robot-assisted laparoscopic adrenalectomy has lower morbidity and shorter hospital stay after the robotic procedures than traditional laparoscopic surgery. In their study, the operation time of different surgical approaches was also compared. The results showed that operation time was similar between the laparoscopic and robotic groups for both lateral transabdominal and posterior retroperitoneal approaches. However, some important perioperative indicators, such as the time of drainage tube removal and postoperative gastrointestinal recovery time, were not involved in their study (13, 14).

In this study, we found that robot-assisted laparoscopic adrenalectomy can significantly reduce intraoperative blood loss and accelerate postoperative gastrointestinal function recovery compared with traditional laparoscopic adrenalectomy. The reason for less bleeding in robot surgery may be that the anatomical structure is clearer when using the robot system, thus avoiding the damage of some small blood vessels. This is very helpful for the clarity of the surgical field (15). Consistent with our findings, Brunaud et al. prospectively evaluated 50 patients with RARLA and 59 patients with RLA, and they found that RARLA was associated with lower blood loss (49.0 vs 71.0 ml, *p* < 0.001) (16). Robotic surgery reduces the damage to surrounding tissues due to clear vision and accurate operation, reduces the damage to the gastrointestinal system, and accelerates the recovery of gastrointestinal function. Lin et al. (17) found that the intestinal recovery time of patients undergoing robotic surgery was faster than that of patients undergoing ordinary laparoscopic surgery, and the anal exhaust and defecation time was shorter. Robotic surgery causes less damage to the patient ‘s body and promotes the recovery of intestinal and other functions.

In the present study, the average operation time of the RARLA group was 30 minutes longer than that of the RLA group. It is generally accepted that the operation time of RARLA is longer than that of RLA in the initial stage of application (18). In fact, some studies have highlighted the docking procedure as the reason for the significant increase in RLA operation time (19). In addition, several variables (robotic operating room, robot platform preparation during anesthesia, and surgical team familiarity with robotic surgery) have a significant effect on the operation time (18). Studies have found that rich laparoscopic surgery experience and previous robotic surgery can significantly reduce the learning curve of RLA (18, 20). The length of hospital stay may be affected by differences in medical reimbursement systems, the distance of patients from referral centers, and cultural expectations. As a new technology, robotic surgery will cost more than traditional laparoscopic surgery, but with the emergence of domestic robots, it is believed that the cost of robotic surgery will be greatly reduced.

In addition, it is worth noting that the choice of surgical approach may also be a factor affecting the outcome of surgery. Previous studies have shown that the retroperitoneal approach has shorter operating time, less bleeding, and earlier recovery of gastrointestinal function compared to the anterior abdominal approach (21–23). Therefore, at present, most patients with adrenal tumors in our center are treated *via* retroperitoneal approach. The anterior abdominal approach is only used in a few large tumors. Based on our experience, we believe that the retroperitoneal approach can reach the adrenal gland with less tissue dissection and avoid peritoneal damage. This may be one reason for less bleeding and faster recovery of gastrointestinal function during operation. Of course, more research is needed.

The main limitation of the study is its retrospective, non-randomized design. The secondary limitation of the study is the small sample size. Due to the limitation of sample size, we cannot analyze the two surgical approaches of lateral transabdominal and posterior retroperitoneal simultaneously. We only study the posterior retroperitoneal approach. The robot system provides a three-dimensional display for surgeons, enhances depth perception, enables surgeons to operate in a comfortable sitting position, keeps eyes, hands and targets consistent, and the device contains a ‘ wrist ‘ joint to improve flexibility. We believe that the ability of robotic surgery to restore hand-eye coordination and three-dimensional vision lost in laparoscopic surgery will enable us to perform complex surgeries with greater accuracy and confidence and better outcomes.

## 5 Conclusion

Compared with traditional laparoscopic adrenalectomy, robot-assisted laparoscopic adrenalectomy can significantly reduce intraoperative blood loss and accelerate postoperative gastrointestinal recovery. It is committed to studying how to reduce the hospitalization time and hospitalization cost of RARLA, which can make RARLA more widely used.

## Data availability statement

The original contributions presented in the study are included in the article/**Supplementary Material**. Further inquiries can be directed to the corresponding authors.

## Ethics statement

The studies involving human participants were reviewed and approved by the Ethical Committee of The First Affiliated Hospital of Nanchang University. The patients/participants provided their written informed consent to participate in this study. Written informed consent was obtained from the individual(s) for the publication of any potentially identifiable images or data included in this article.

## Author contributions

Conception and design: GW and XZ. Surgeons: GW and XZ. Acquisition of data: SX. Preparation of tools: YY and WL. Analysis and interpretation of data: XL. Drafting of the manuscript and statistical analysis: XL and SX. Critical revision: GW and HX. Obtaining funding: XZ and HX. All authors contributed to the article and approved the submitted version.

## References

[1] Gagner M Lacroix A Bolté E. Laparoscopic adrenalectomy in cushing's syndrome and pheochromocytoma. New Engl J Med (1992) 327(14):1033. doi: 10.1056/nejm199210013271417 1387700

[2] Smith CD Weber CJ Amerson JR. Laparoscopic adrenalectomy: New gold standard. World J Surg (1999) 23(4):389–96. doi: 10.1007/pl00012314 10030863

[3] Haglind E Carlsson S Stranne J Wallerstedt A Wilderäng U Thorsteinsdottir T. Urinary incontinence and erectile dysfunction after robotic versus open radical prostatectomy: A prospective, controlled, nonrandomised trial. Eur Urol (2015) 68(2):216–25. doi: 10.1016/j.eururo.2015.02.029 25770484

[4] Bochner BH Dalbagni G Sjoberg DD Silberstein J Keren Paz GE Donat SM. Comparing open radical cystectomy and robot-assisted laparoscopic radical cystectomy: A randomized clinical trial. Eur Urol (2015) 67(6):1042–50. doi: 10.1016/j.eururo.2014.11.043 PMC4424172 25496767

[5] Pineda-Solís K Medina-Franco H Heslin MJ. Robotic versus laparoscopic adrenalectomy: A comparative study in a high-volume center. Surg endoscopy (2013) 27(2):599–602. doi: 10.1007/s00464-012-2496-9 22955998

[6] Brandao LF Autorino R Laydner H Haber GP Ouzaid I De Sio M. Robotic versus laparoscopic adrenalectomy: A systematic review and meta-analysis. Eur Urol (2014) 65(6):1154–61. doi: 10.1016/j.eururo.2013.09.021 24079955

[7] Heger P Probst P Hüttner FJ Gooßen K Proctor T Müller-Stich BP. Evaluation of open and minimally invasive adrenalectomy: A systematic review and network meta-analysis. World J Surg (2017) 41(11):2746–57. doi: 10.1007/s00268-017-4095-3 28634842

[8] Horgan S Vanuno D. Robots in laparoscopic surgery. J laparoendoscopic advanced Surg techniques Part A (2001) 11(6):415–9. doi: 10.1089/10926420152761950 11814134

[9] Brandao LF Autorino R Zargar H Krishnan J Laydner H Akca O. Robot-assisted laparoscopic adrenalectomy: Step-by-Step technique and comparative outcomes. Eur Urol (2014) 66(5):898–905. doi: 10.1016/j.eururo.2014.04.003 24830625

[10] Gan L Peng L Li J Meng C Li K Wu J. Comparison of the effectiveness and safety of robotic-assisted and laparoscopic in adrenalectomy: A systematic review and meta-analysis. Int J Surg (London England) (2022) 105:106853. doi: 10.1016/j.ijsu.2022.106853 36075556

[11] Agcaoglu O Aliyev S Karabulut K Mitchell J Siperstein A Berber E. Robotic versus laparoscopic resection of Large adrenal tumors. Ann Surg Oncol (2012) 19(7):2288–94. doi: 10.1245/s10434-012-2296-4 22396002

[12] Karabulut K Agcaoglu O Aliyev S Siperstein A Berber E. Comparison of intraoperative time use and perioperative outcomes for robotic versus laparoscopic adrenalectomy. Surgery (2012) 151(4):537–42. doi: 10.1016/j.surg.2011.09.047 22142558

[13] Niglio A Grasso M Costigliola L Zenone P De Palma M. Laparoscopic and robot-assisted transperitoneal lateral adrenalectomy: A Large clinical series from a single center. Updates Surg (2020) 72(1):193–8. doi: 10.1007/s13304-019-00675-8 31473921

[14] De Crea C Pennestrì F Voloudakis N Sessa L Procopio PF Gallucci P. Robot-assisted vs laparoscopic lateral transabdominal adrenalectomy: A propensity score matching analysis. Surg endoscopy (2022) 36(11):8619–29. doi: 10.1007/s00464-022-09663-3 PMC9613740 36190555

[15] Makay O Erol V Ozdemir M. Robotic adrenalectomy. Gland Surg (2019) 8(Suppl 1):S10–s6. doi: 10.21037/gs.2019.01.09 PMC6646813 31404190

[16] Brunaud L Bresler L Ayav A Zarnegar R Raphoz AL Levan T. Robotic-assisted adrenalectomy: What advantages compared to lateral transperitoneal laparoscopic adrenalectomy? Am J Surg (2008) 195(4):433–8. doi: 10.1016/j.amjsurg.2007.04.016 18304514

[17] Lin Y Zhang Y Luo L Zhang X. Clinical effect of robot-assisted radical cystectomy in bladder cancer. Am J Trans Res (2021) 13(9):10545–53. PMC8506996 34650725

[18] Mihai R Donatini G Vidal O Brunaud L. Volume-outcome correlation in adrenal surgery-an eses consensus statement. Langenbeck's Arch Surg (2019) 404(7):795–806. doi: 10.1007/s00423-019-01827-5 31701230 PMC6908553

[19] Hyams ES Stifelman MD. The role of robotics for adrenal pathology. Curr Opin Urol. (2009) 19(1):89–96. doi: 10.1097/MOU.0b013e32831b446c 19057223

[20] Ludwig WW Gorin MA Pierorazio PM Allaf ME. Frontiers in robot-assisted retroperitoneal oncological surgery. Nat Rev Urol (2017) 14(12):731–41. doi: 10.1038/nrurol.2017.149 28895564

[21] Gavriilidis P Camenzuli C Paspala A Di Marco AN Palazzo FF. Posterior retroperitoneoscopic versus laparoscopic transperitoneal adrenalectomy: A systematic review by an updated meta-analysis. World J Surg (2021) 45(1):168–79. doi: 10.1007/s00268-020-05759-w 32856097

[22] Conzo G Tartaglia E Gambardella C Esposito D Sciascia V Mauriello C. Minimally invasive approach for adrenal lesions: Systematic review of laparoscopic versus retroperitoneoscopic adrenalectomy and assessment of risk factors for complications. Int J Surg (London England) (2016) 28 Suppl 1:S118–23. doi: 10.1016/j.ijsu.2015.12.042 26708860

[23] Lairmore TC Folek J Govednik CM Snyder SK. Improving minimally invasive adrenalectomy: Selection of optimal approach and comparison of outcomes. World J Surg (2016) 40(7):1625–31. doi: 10.1007/s00268-016-3471-8 26932878

